# Data Delivery Method Based on Neighbor Nodes' Information in a Mobile Ad Hoc Network

**DOI:** 10.1155/2014/793171

**Published:** 2014-02-04

**Authors:** Shigeru Kashihara, Takuma Hayashi, Yuzo Taenaka, Takeshi Okuda, Suguru Yamaguchi

**Affiliations:** ^1^Graduate School of Information Science, Nara Institute of Science and Technology, Takayama, Ikoma, Nara 8916-5, Japan; ^2^Information Technology Center, The University of Tokyo, Yayoi, Bunkyo, Tokyo 2-11-16, Japan

## Abstract

This paper proposes a data delivery method based on neighbor nodes' information to achieve reliable communication in a mobile ad hoc network (MANET). In a MANET, it is difficult to deliver data reliably due to instabilities in network topology and wireless network condition which result from node movement. To overcome such unstable communication, opportunistic routing and network coding schemes have lately attracted considerable attention. Although an existing method that employs such schemes, MAC-independent opportunistic routing and encoding (MORE), Chachulski et al. (2007), improves the efficiency of data delivery in an unstable wireless mesh network, it does not address node movement. To efficiently deliver data in a MANET, the method proposed in this paper thus first employs the same opportunistic routing and network coding used in MORE and also uses the location information and transmission probabilities of neighbor nodes to adapt to changeable network topology and wireless network condition. The simulation experiments showed that the proposed method can achieve efficient data delivery with low network load when the movement speed is relatively slow.

## 1. Introduction

Despite their widespread use, smart mobile devices (SMDs) still carry out communications only through existing wireless infrastructures, such as base stations. However, since SMD density is high in large cities, there are many more opportunities for SMDs to directly communicate with each other. Such communication has the potential to make a wireless network more resilient. In the near future, SMDs will directly and autonomously connect to each other to form a mobile ad hoc network (MANET). MANETs will also coexist with or replace current wireless infrastructures.

Compared with the existing wireless infrastructure, since a MANET requires no cost for network design, construction, and management, it furthermore has the potential to improve fault tolerance by having a distributed control. However, it is difficult for a MANET to make reliable communication among moving nodes due to the instability of the network topology and the wireless network condition. In particular, the movement of nodes characterizing such a wireless network exacerbates this difficulty. To provide reliable communication, a MANET thus requires a reliable data delivery method that can adapt to a changeable network topology and an unstable wireless network.

To improve data delivery over an unstable wireless network, Biswas and Morris [2] proposed opportunistic routing, which uses packet distribution via wireless broadcast. In this approach, multiple neighbor nodes (NNs) simultaneously receive a packet transmitted by a sender, and they then help to forward the packet to increase throughput. On the other hand, to enhance the efficiency of data delivery, network coding schemes are also receiving increasing attention [3]. In a network coding scheme, in addition to a conventional packet-forwarding process, some packets are coded by a sender or routers and transmitted as a single-coded packet. A receiver can then decode the coded packets when it receives enough of them. Consequently, since each coded packet randomly has a random linear combination of multiple packets, the communication becomes more resilient against packet loss and the reliability of data delivery is improved. Based on the previous two approaches, and to further improve the efficiency of data delivery in a fixed wireless mesh network, Chachulski et al. proposed MAC-independent opportunistic routing and encoding (MORE) method [1]. MORE combines opportunistic routing and network coding to enhance data delivery and to reduce the number of redundant packets that arise during opportunistic routing. Although MORE performs well in an unstable wireless mesh network, it is difficult to adapt to the movement of nodes because each node needs to share all link conditions throughout the entire network. Delivery of data among moving nodes therefore remains a challenging issue, because a MANET can have no static route.

To improve reliable communication in a MANET, we now propose a data delivery method that employs opportunistic routing, network coding, and information about NNs. Opportunistic routing and network coding technologies are the same as those in MORE, since we use MORE as a base system. However, in a MANET, since each node moves freely, it is impossible to accurately grasp the entire network topology and wireless network condition, as is the case with MORE. Therefore, in the proposed method, the following new functions are added to MORE to adapt to the changeable network topology and wireless network condition. First, each node periodically broadcasts an information packet that includes its own and the NNs' location information, and it then obtains the packet error rate (PER) to the one-hop NN from the bit error rate (BER) of the received information packet. Next, the source node (SN) and relay nodes (RNs) efficiently distribute coded packets to the next RNs according to the location information and transmission probability of their NNs. Whereas in MORE an SN computes a forwarders list from the SN to the destination node (DN), in our proposed method each node makes a forwarders list for only the next RNs hop-by-hop. The primary contribution of the present study is thus efficient data distribution using network coding and opportunistic routing based on NNs' information, that is, location information and PER. Through the simulation experiments, we demonstrate the basic characteristics of the proposed method in a MANET.

The remainder of the present paper is organized as follows.  Section 2 presents related work.  Section 3 proposes a data delivery method using opportunistic routing, network coding, and the information of NNs for a MANET.  Section 4 evaluates the communication performance of the proposed method. Finally, concluding remarks are presented in Section 5.

## 2. Related Work

As mentioned above, opportunistic routing and network coding have been proposed to improve the efficiency of data delivery in an unstable wireless mesh network. Since the method proposed herein is based on these concepts, we outline these approaches in Sections 2.1 and 2.2; Section 2.3 then describes issues related to data delivery in a MANET.

### 2.1. Opportunistic Routing

Various routing protocols have been proposed for a MANET [4–7]. In these protocols, data delivery to a DN is based on a routing table. This approach is satisfactory when the channel quality is very good and nodes are always on but may otherwise be very wasteful. Indeed, in a dynamic network such as a MANET, maintaining a reliable routing table is a particularly difficult task.

As a routing method suited to such an unstable wireless network, opportunistic routing has lately attracted considerable attention [1, 2, 8, 9]. Opportunistic routing has been introduced by means of the ExOR protocol [2]. Since opportunistic routing exploits wireless broadcasting, multiple NNs receive a packet transmitted by a sender, at the same time, and candidate forwarders specified in the packet rebroadcast the packet. As a result, ExOR can increase the transmission probability in a wireless mesh network.

**Figure 1:**
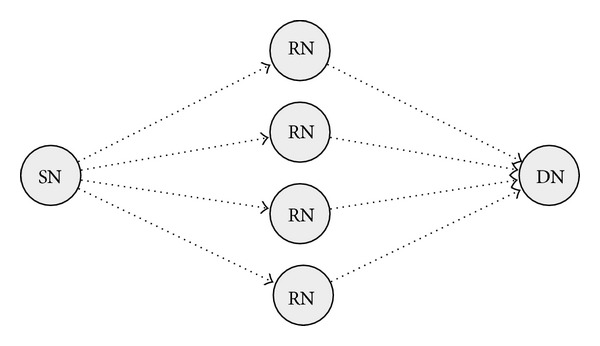
Advantage of opportunistic routing.

Figure 1 illustrates the basic advantage of opportunistic routing. If the transmission probability of each link is *p* (0 < *p* ≤ 1), then the transmission probability from the SN to the DN is *p* ^2^ in unicast transmission. On the other hand, if all four intermediate nodes, that is, RNs, can relay a packet by opportunistic routing, then the probability of transmission to the DN is improved to 1 − (1 − *p* ^2^)^4^.

### 2.2. Network Coding

**Figure 2:**
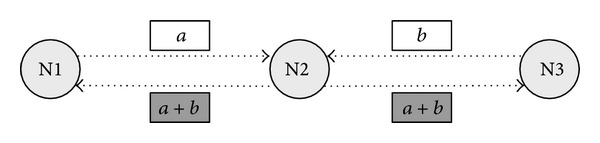
Advantage of network coding over a wireless network.

Ahlswede et al. proposed a method to improve the transmission the transmission probability by coding some packets over a network, that is, network coding [3].  Figure 2 outlines the basic advantage of network coding over a wireless network. We assume that nodes 1 (N1) and 3 (N3) communicate with each other. If network coding is not employed, N1 first sends a packet (*a*) to node 2 (N2) and N2 then forwards the packet to N3. N3 also sends a packet (*b*) to N2, which is then forwarded to N1. Thus, the transmission count is four. If network coding is employed, after receiving packets (*a*) and (*b*), N2 broadcasts a coded packet (*a* + *b*) to N1 and N3. N1 and N3 can then extract the necessary packet from the coded packet by themselves. Therefore, the transmission count is reduced to three.

Based on this concept, a number of network coding schemes over wireless networks have been proposed [1, 10, 11]. In COPE [10], as described above, when two sender nodes transmit packets, the intermediate node XORs their packets and transmits the single-coded packet to them. If the two receiver nodes have sufficient information to decode the coded packet, the packet is then decoded. CodeCast [11] is a network-coding-based multicast protocol for an ad hoc network and employs a random network coding to implement both localized loss recovery and path diversity transparently. Simulation results demonstrated that CodeCast achieves an almost perfect packet delivery ratio while maintaining a lower overhead than conventional multicast. In the present study, to improve the data delivery in unicast communication, we focus on MORE [1], which improves the transmission probability by opportunistic routing and network coding. MORE codes and decodes together only packets belonging to the same flow, that is, an intraflow technique, to obtain higher throughput. In addition, MORE implements both opportunistic routing and intraflow network coding in a real system.

**Figure 3:**
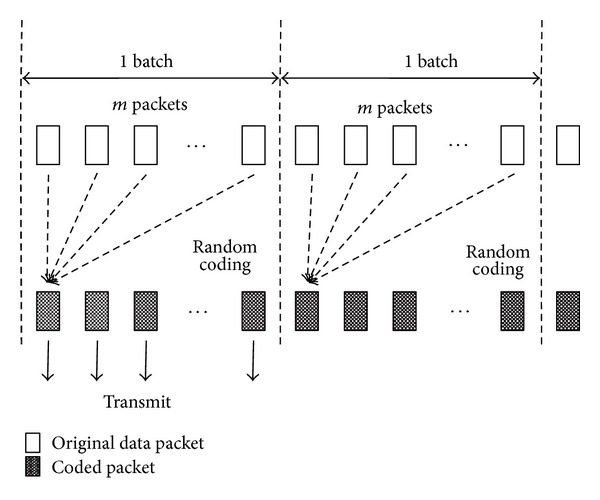
Random network coding in MORE.

We now briefly explain the operation of MORE, which is a base system in the proposed method. As illustrated in Figure 3, to provide reliable file transfer, the SN generates batches from the file which are divided into *m* packets each. The SN then broadcasts a coded packet created by a random linear combination of *m* native packets (uncoded packets are referred to as native packets) in the current batch. If an RN receives an innovative packet (coded packets with new information are referred to as innovative packets), it also creates and broadcasts a random linear combination of the coded packets that were received in the RN's buffer. If the received coded packet is not an innovative packet, the RN discards it because the coded packet has no new information. On the other hand, when the DN receives a coded packet, it also checks whether the received coded packet is an innovative packet. If it is not, the DN discards the coded packet because it contains no new information to decode the batch. Conversely, if the DN receives *m* innovative packets, it can decode the entire batch; whereupon, to obtain the next batch, the DN sends back an ACK packet to the SN. Therefore, since it is more likely that coded packets broadcasted by the SN contain information that is different from the coded packets broadcasted by an RN, MORE can also alleviate the problem of redundant packets that arise due to opportunistic routing. Moreover, MORE can achieve reliable communication because it controls data transmission at every batch, such as a retransmission mechanism.

### 2.3. Data Delivery Issues in a MANET

Since the network topology and wireless network condition in a MANET are changeable due to the movement of nodes, the improvement of transmission efficiency is an important issue. Many routing protocols for MANET have been studied to improve data delivery [7]. However, they do not have reliable transmission controls such as a retransmission mechanism, so they cannot achieve reliable communication. For instance, since traditional MANET routing protocols such as DSR [5] and AODV [4] typically need to discover a route before exchanging packets between communication nodes, the predetermined routes are susceptible to changeable link quality or link failure when nodes move freely. To alleviate such impacts on routes, position-based routings have been proposed [12]. Moreover, approaches that combine position-based routing with opportunistic routing [13, 14] can also enhance the delivery ratio of position-based routing. These approaches select nodes that are closest to the destination from among all the current node's neighbors as the next forwarding nodes, without considering transmission probability to the next forwarding nodes. However, if transmission probabilities to all the forwarding nodes are severely low, the sent data may be lost and the delivery ratio may be reduced, because opportunistic routing has no retransmission mechanism. To make reliable communication, we therefore need to have a retransmission mechanism.

As described in Section 2.2, since MORE controls data transmission at every batch, it can achieve a reliable communication over unstable wireless mesh networks. To optimize transmission efficiency, MORE employs expected transmission count (ETX) [15]; the ETX of a link is calculated from the forward and reverse delivery ratio of the link. Since each node measures the transmission probability to communicable NNs by periodically sending ICMP packets, each ETX can be calculated from the measurement results. All of the ETXs are then shared throughout the entire network. Before sending a packet, the SN uses the ETX values to make a forwarders list, which specifies the RNs that will forward the packet from the SN to the DN, to limit unnecessary forwarding of packets by RNs. Since MORE focuses on a fixed wireless mesh network, it is easy to share ETXs over the entire network because the transmission probability does not change frequently or abruptly. In addition, routing paths based on ETXs do not change drastically. However, in a MANET, the network topology and ETX values change substantially due to the constant movement of nodes. Consequently, MORE cannot avoid communication degradation in a MANET because all of the ETXs in the entire network cannot be quickly updated due to frequent changes in the network topology and wireless network condition. Therefore, to achieve reliable communication over a MANET, we need to quickly update the wireless network condition of communicable NNs and select appropriate RNs.

## 3. Data Delivery Method

To enhance the efficiency of data delivery in a MANET, we first employ the same opportunistic routing and network coding used in MORE. However, as described in Section 2.3, MORE has difficulty in appropriately delivering packets based on ETXs due to the changeable network topology and wireless network condition. Therefore, in the present study, to adapt to the changeable network topology and wireless network condition, we propose a data delivery method based only on NNs' information, that is, location information and PER of NNs. Since the proposed method does not need to share ETXs over the entire network, it can quickly update the wireless network condition of NNs and appropriately select RNs hop-by-hop. In this section, we first outline the proposed method in Section 3.1 and then explain its operations in detail in Sections 3.2, 3.3, and 3.4.

### 3.1. Operation Outline

In a MANET, it is impossible to obtain a completely stable route from an SN to a DN based on ETXs due to the changeable network topology and wireless network condition. In addition, a long time is required to measure and share ETXs. We now consider a data delivery method based only on NNs' information, rather than sharing ETXs over the entire network.

**Figure 4:**
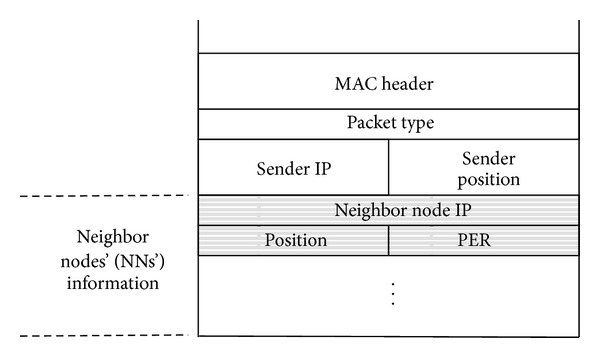
Format of an information packet.

**Figure 5:**
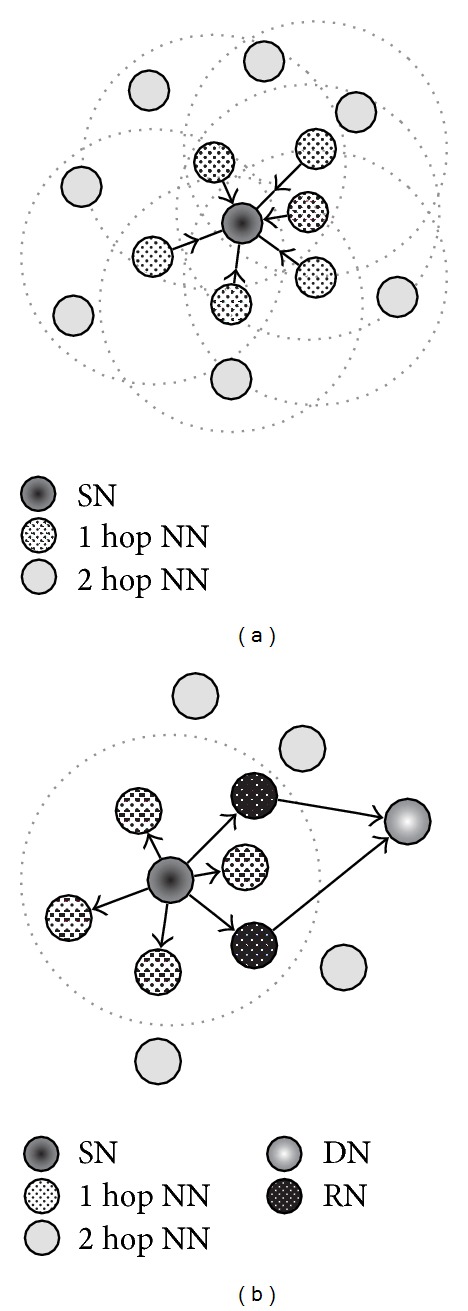
Outline of the data delivery process.

In our proposed method, first of all, to obtain NNs' information, each node periodically broadcasts an information packet and collects its own NNs' information by receiving information packets that NNs broadcast.  Figure 4 shows the information packet format. PACKET TYPE field indicates that the packet is an information packet, while SENDER IP and SENDER POSITION fields indicate the IP address and geographical position of the sender node, respectively. The packet also includes NN's information that the sender already knows, consisting of an IP address, a geographical position, and PER between the sender and the NN. We assume that each node can obtain location information using a positioning system, such as GPS. The receiver can also obtain the PER to the sender from the BER of the received information packet. Thus, as illustrated in Figure 5(a), each node can obtain the other nodes' location information and PER within two hops.

**Figure 6:**
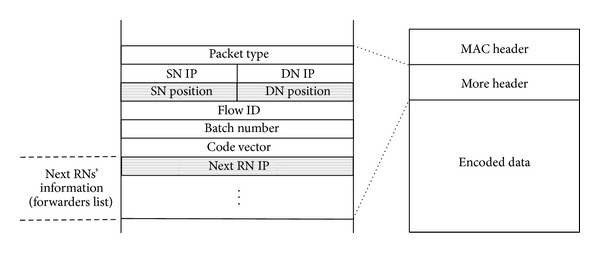
Format of a coded packet.

Next, to efficiently deliver data, the SN first generates a forwarders list, which only specifies the next RNs that forward a coded packet by broadcast. Note that the next RNs are selected based on node location and transmission probability for the one-hop NNs. The SN then broadcasts the coded packet including the forwarders list (see Figure 5(b)).  Figure 6 illustrates the coded packet format, which is based on a MORE coded packet; the grey fields indicate additional fields in our proposed method. PACKET TYPE field indicates that the packet is a coded packet. Since data delivery uses location information, the coded packet includes source and destination's geographical positions, that is, SN POSITION and DN POSITION fields. In MORE a coded packet holds all forwarder nodes' information from an SN to a DN in a forwarders list, while our proposed method inserts only the next RNs' information into the forwarders list because it is difficult to maintain a stable route from an SN to a DN. Thus, the forwarders list is updated in a hop-by-hop fashion, and its size depends on the number of NNs that forward the packet. In nodes that receive the coded packet, the nodes specified in the forwarders list become RNs. The RNs also use the same operation as the SN to select the next RNs and forward the coded packet after replacing the previous forwarders list with a new one. Thus, the operation is repeated until the coded packet reaches the DN. In the following sections, we explain in detail the operations used to achieve efficient data delivery in a MANET.

### 3.2. Neighbor Node Information

As noted above, it is difficult to maintain a stable transmission path in a MANET. The proposed method therefore forwards a coded packet by broadcasting it in a hop-by-hop fashion. To achieve this, the information of NNs is indispensable. In the proposed method, to obtain the NNs' information, each node, say node *i*, periodically broadcasts an information packet that includes the location of node *i*, and the locations and the PER of the NNs that node *i* already knows, as described in the previous section. Each node can also obtain a PER calculated from the BER of the information packet as the transmission probability to a one-hop NN. The PER is calculated by the following equation:

(1) $$\begin{array}{c} \text{PER}=1-{\left(1-\text{BER}\right)}^{L}, \end{array}$$

where *L* indicates the packet size. Thus, each node can quickly and reliably obtain the PER to one-hop NNs, and then each node makes a routing table containing the location information and the PER.

### 3.3. Selection of RNs

Since the proposed method forwards a coded packet in broadcast transmission, if we employ no transmission control, the packet would be distributed in all directions by every NN. This would lead to the consumption of unnecessary wireless resources, and the communication quality of the entire network would degrade. Therefore, to minimize the unnecessary consumption of wireless resources, each node that broadcasts a coded packet must generate a forwarders list based on location information and transmission probability for NNs.

**Figure 7:**
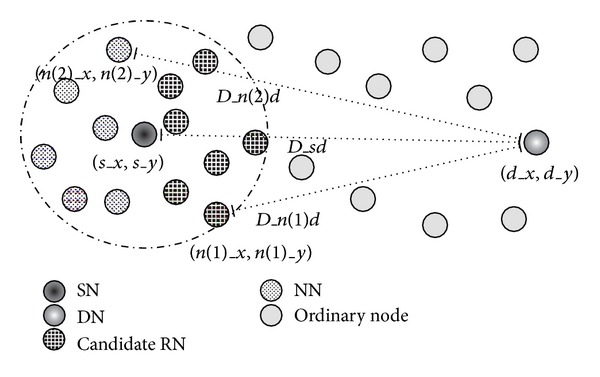
Selection of candidate RNs.

We explain here the process of generating a forwarders list for an SN. Note that RNs also use the same process. To make a communication with the DN, the SN must first obtain the location information of the DN. However, in the present paper, we focus on data delivery because the problem is almost as difficult in either case. Thus, we assume, for example, that the SN can obtain the location information of the DN by broadcasting a message such as AODV [4]. After obtaining the location information of the DN (*d*_*x*_, *d*_*y*_), the SN calculates the distance to the DN (*D*_*sd*_) by the following equation:

(2) $$\begin{array}{c} D_{sd}=\sqrt{{\left(d_{x}-s_{x}\right)}^{2}+{\left(d_{y}-s_{y}\right)}^{2}}, \end{array}$$

where (*s*_*x*_, *s*_*y*_) indicates the location information of the SN. Next, in order to make a forwarders list, the SN also calculates the distances between each one-hop NN (*n*_*x*_, *n*_*y*_) in its own routing table and the DN (*D*_*nd*_). Then, based on the following equation, the SN selects NNs that are closer to the DN than the SN as candidate RNs:

(3) $$\begin{array}{c} N=\left\{n\;|\;n\in D_{sd}>D_{nd}\right\}, \end{array}$$

where *N* is a set of candidate RNs. Thus, this process can provide the direction in order to deliver packets to the DN (see Figure 7). In this way, although packets can approach the DN, reachability is not guaranteed because no consideration is given to the communication quality between nodes. In addition, selecting more RNs leads to an increase in network load. The proposed method thus needs to restrict RNs that forward coded packets by considering the transmission probability to candidate RNs.

As described in Section 3.2, we employ PER as a metric of communication quality to NNs. A sender selects some RNs with high PER to enhance the transmission range and to conserve wireless resources. If the sender simply selects a number of RNs with low PER, wireless resources are unnecessarily consumed because the number of hops to the DN increases. Hence, since there is a trade-off between PER and the number of hops to the DN, the proposed method selects RNs with high PER.

**Figure 8:**
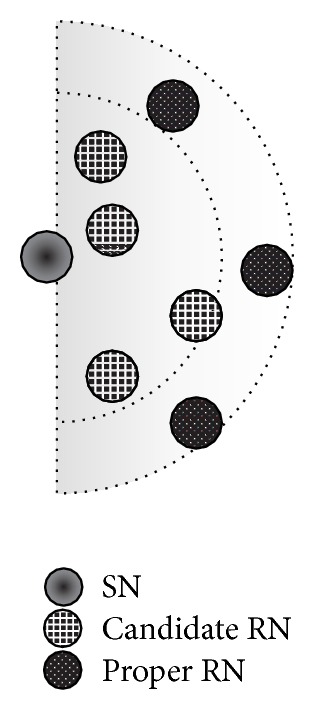
Selection of proper RNs.

From the candidate RNs (*N*), obtained from (3), we determine the appropriate RNs to actually forward a coded packet (see Figure 8). To obtain a transmission probability greater than a certain value (*α*_*t*_), we select RNs according to the following equation:

(4) $$\begin{array}{c} 1-\prod_{N}\text{PE}{\text{R}}_{sn}>\alpha_{t}, \end{array}$$

where *s* indicates the SN and *n* is a candidate RN, selected in descending order of PER. Candidate RNs are selected until (4) is satisfied or all candidate RNs have been selected. By selecting multiple candidate RNs, the transmission probability is adjusted to *α*_*t*_ or higher. In this way, the SN specifies the next proper RNs in the forwarders list and then forwards a coded packet with the forwarders list. Therefore, when the NNs receive the coded packet that the SN broadcasted, only the NNs specified in the forwarders list become RNs. These RNs then broadcast the coded packet after selecting the next RNs, just as the SN did.

### 3.4. Updating of DN Location Information by RNs

In MORE, the only packet that the SN can receive from the DN is an ACK packet, which is sent after the DN has decoded an entire batch. In the proposed method, if the SN was able to update the location information of the DN only when the SN received an ACK packet from the DN, then the SN might hold the erroneous location information of the DN for a long time. In the worst situation, the SN might not be able to deliver packets to the DN if the DN had moved out of RNs' reach. Therefore, to enhance the data delivery to the DN, RNs within two hops from the DN update the location information of the DN to the latest location information based on the NNs' information.

**Figure 9:**
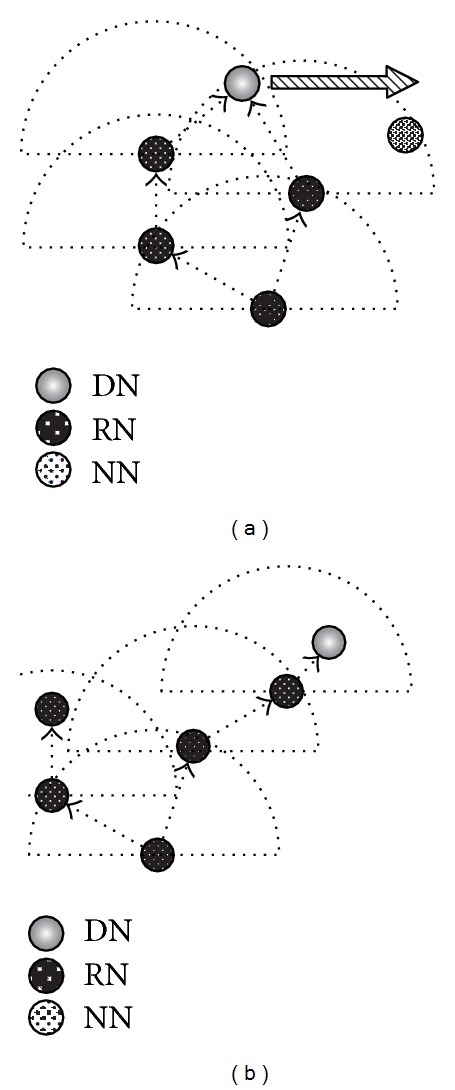
Updating of the DN location information by RNs.

The location information update of DN works as follows. As illustrated in Figure 9(a), when an RN receives a coded packet, it normally selects the next RNs based on its own NN's information and the location information of the DN in the packet header and forwards the coded packet. At this time, if the DN moves, the actual location information of the DN is different from that in the packet header. To alleviate that problem, in the proposed method, if an RN within two hops from the DN has the actual location information of the DN in its own routing table, the RN uses the information in the routing table to select the next RNs and also updates the location information of the DN in the packet header to the information in the routing table. Since each node broadcasts an information packet periodically (e.g., every second), NN's information is considered to contain the latest location information of the DN. Thus, as illustrated in Figure 9(b), even if the SN sends a coded packet including erroneous location information of the DN, the packet has a fair possibility of reaching the DN because RNs within two hops from the DN can update the location information of the DN. On the other hand, if an RN has not recorded the changed location information of the DN in its routing table, it deals with a coded packet based on the erroneous location information in the packet header. Consequently, if all RNs lose track of the DN, the SN may need to rediscover the DN.

## 4. Simulation Experiments

This section demonstrates the communication performance of the proposed method in a MANET through simulation experiments using Qualnet 4.5.1 [16].  Section 4.1 investigates the basic communication performance of the proposed method and MORE. Sections 4.2, 4.3, and 4.4, respectively, describe the communication performance of the proposed method for the cases in which the DN, the SN, and the RNs move. In addition, Section 4.5 investigates the communication performance for the case in which the SN, DN, and RNs move.

### 4.1. Communication Performance of The Proposed Method and MORE

**Figure 10:**
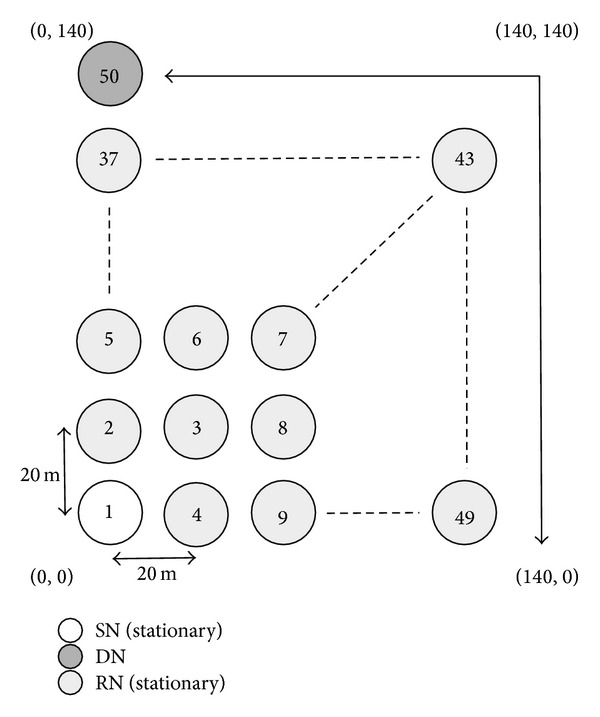
Simulation model 1 (movement of DN).

This section provides the basic communication performance of the proposed method and MORE in the case when the DN moves at 1 m/s. We first consider the simulation model illustrated in Figure 10, in which 50 nodes are located at regular intervals of 20 m over a 140 meter squared area. All nodes are assumed to be equipped with an IEEE 802.11 g wireless interface of a fixed 54 Mb/s transmission rate. In the simulation experiments, we investigate the communication performance for one flow. In one flow, one batch contains 64 coded packets, each of which consists of 1,500 bytes, and a coded packet is sent every second. One simulation experiment ends when the DN successfully decodes five batches from the SN; that is, the DN must have received at least 320 coded packets. Also, during the experiment, each node also broadcasts an information packet every second.

We now evaluate the communication quality for cases in which the DN moves at 1 m/s. In Figure 10, the SN of node 1, situated at (0, 0), communicates with the DN of node 50, situated at (0, 140). The DN continues to move at 1 m/s according to the following directions: (0, 140) → (140, 140) → (140, 0) → (140, 140) → (0, 140). The remaining nodes are all stationary. To demonstrate the effectiveness of the NNs' information, we investigate the communication performance of the proposed method without location information updating of the DN by RNs. Note that, in the simulation, the SN can update the location information of the DN only when it receives an ACK packet from the DN.

**Figure 11:**
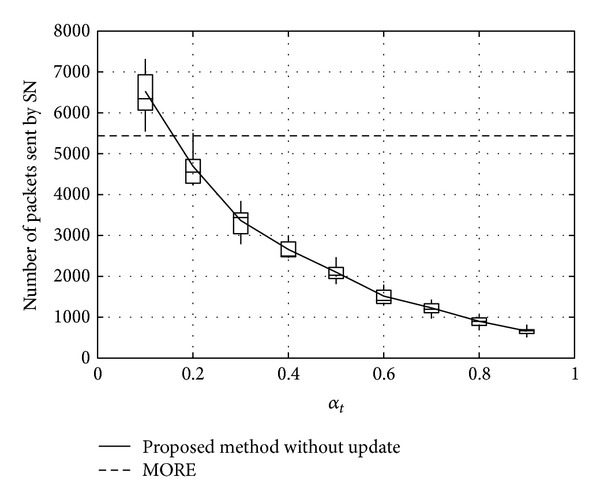
Number of packets sent by the SN.

**Figure 12:**
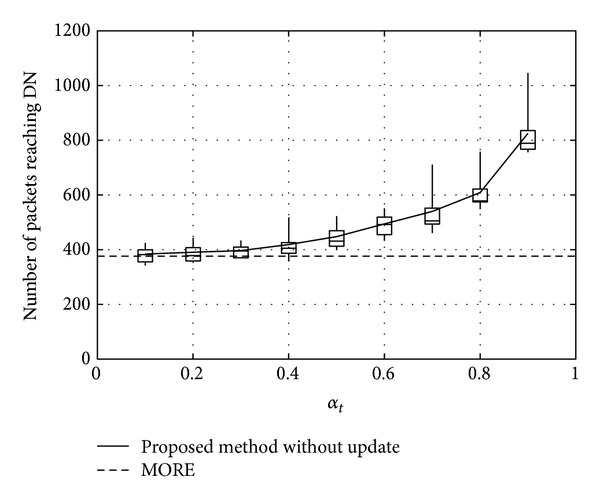
Number of packets reaching the DN.

**Figure 13:**
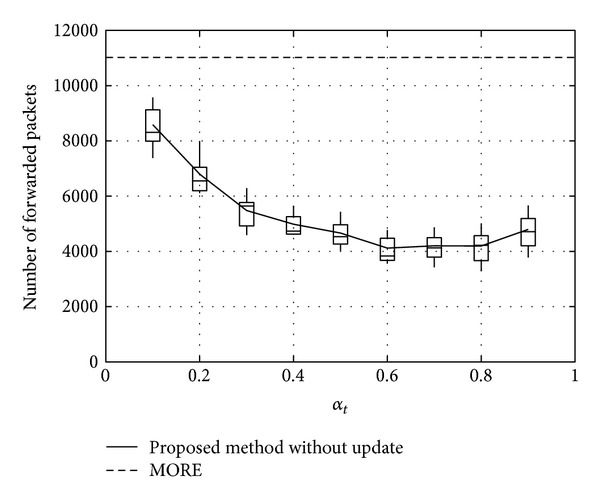
Number of packets forwarded over the network.

Figures 11, 12, and 13 show the number of packets sent by the SN, the number of packets that reached the DN, and the number of packets forwarded over the entire network, respectively, for *α*_*t*_, in the case when the DN moves at 1 m/s. Note that, in all the figures, the term “packets” means all the coded packets including innovative and noninnovative packets. In these figures, since the original MORE does not employ *α*_*t*_, a value obtained for MORE in the simulation mode is employed for all *α*_*t*_. These figures indicate that the number of packets sent by the SN decreases with increasing *α*_*t*_, while the number of packets reaching the DN increases. This is because, as the SN and RNs select more RNs with increasing *α*_*t*_, the number of packets sent by the SN decreases. Moreover, since the higher number of RNs enhances the transmission probability, the number of packets reaching the DN is also increased. Compared with MORE, which cannot appropriately update the NNs' information (i.e., ETXs), the proposed method provides better performance. In Figure 13, the number of forwarded packets tends to initially decrease with increasing *α*_*t*_ but then increases again when *α*_*t*_ exceeds 0.8. This is because the increase in the number of selected RNs, that is, the increased number of hops to the DN, impacts the network load. Particularly, since unnecessary transmissions consume limited wireless resources and lead to performance degradation in a wireless network, we need to reduce the network load as much as possible and make more packets reach the DN. Thus, since an optimal value for *α*_*t*_ is between 0.6 and 0.8, *α*_*t*_ is set to 0.7 in the present study.

### 4.2. Communication Performance for Movement of DN

In the previous section, we investigated how the movement of the DN impacts the proposed method when the location of the DN is not updated by RNs. This section evaluates the performance of the proposed method with location updating by RNs for various movement speeds (i.e., from 1 m/s to 17 m/s) of the DN. The DN moves as described in Section 4.1, and the remaining nodes are all stationary.

**Figure 14:**
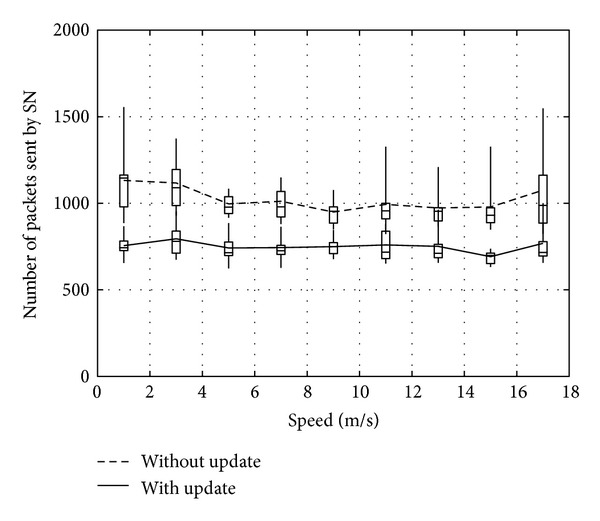
Number of packets sent by the SN.

**Figure 15:**
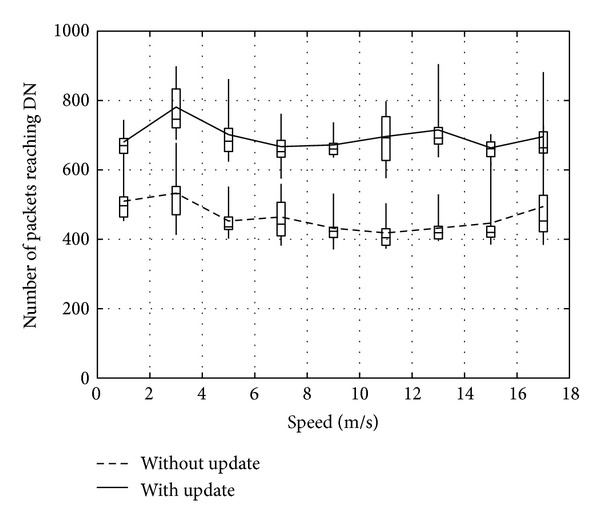
Number of packets reaching the DN.

**Figure 16:**
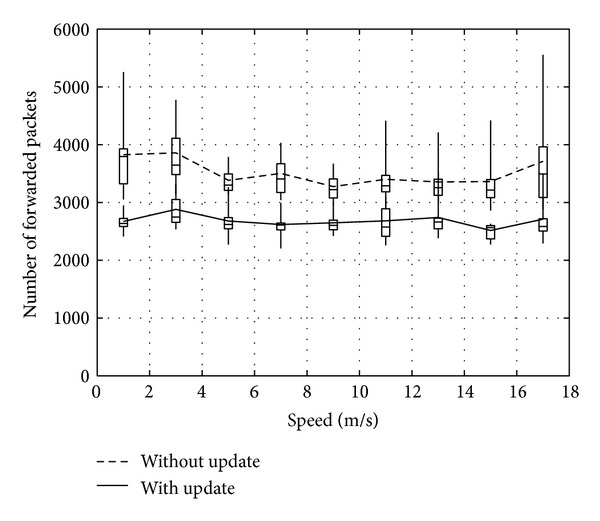
Number of packets forwarded over the network.

Figures 14, 15, and 16 show the number of packets sent by the SN, the number of packets that reached the DN, and the number of packets forwarded over the entire network, respectively. The evaluation compares the performance of the proposed method with and without DN's location being updated by RNs. From the results, the method with DN's location being updated by RNs provides better performance irrespective of the movement speed of the DN. Therefore, the method reduces the number of packets sent by the SN, while the DN receives more packets. As a result, the network load is also improved substantially.

### 4.3. Communication Performance for Movement of SN

**Figure 17:**
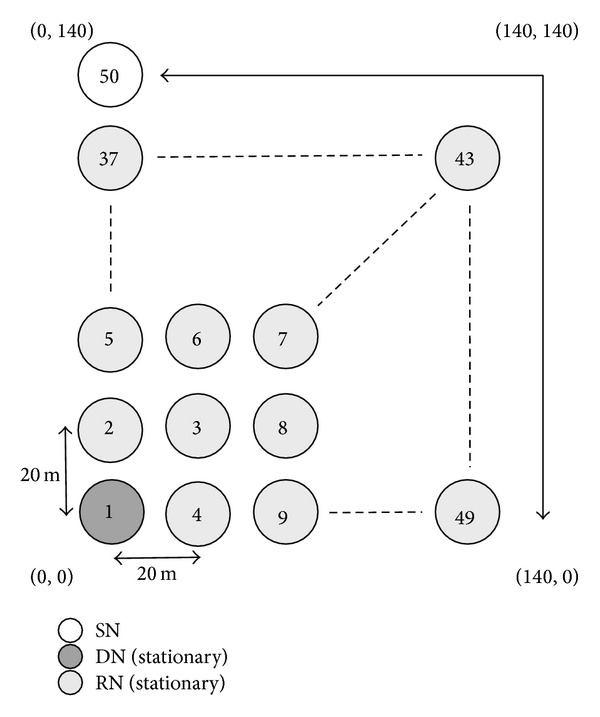
Simulation model 2 (movement of SN).

This section investigates how SN's movement speed (from 1 m/s to 17 m/s) affects the communication performance of the proposed method with location updating by RNs. As illustrated in Figure 17, the SN of node 50 sends five batches to the DN of node 1. The SN moves between (0, 140) and (140, 0), as did the DN in the previous section, and then the DN and RNs are all stationary.

**Figure 18:**
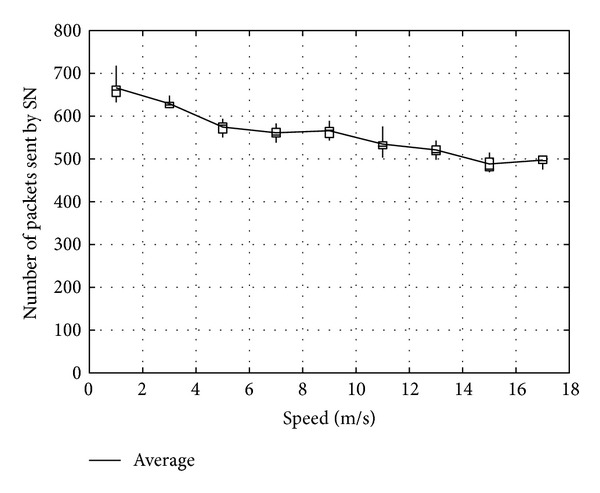
Number of packets sent by the SN.

**Figure 19:**
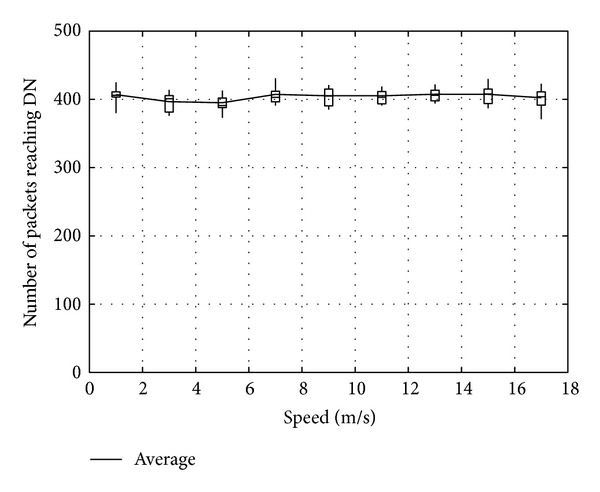
Number of packets reaching the DN.

**Figure 20:**
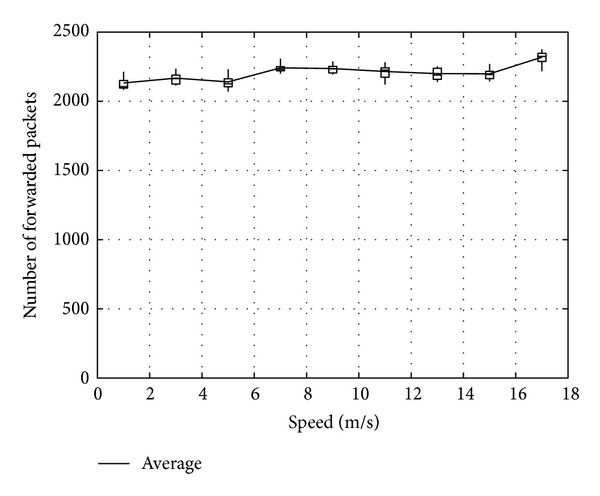
Number of packets forwarded over the network.

Figures 18, 19, and 20 show the number of packets sent by the SN, the number of packets that reached the DN, and the number of packets forwarded over the entire network, respectively. As the speed of the SN increases, the number of packets it sends decreases, while the network load increases. On the other hand, the number of packets that reaches the DN is not significantly changed. Now, let us consider the reasons for these results. In the simulation, the positions of the DN and RNs do not change. Thus, the performance degradation with changes in speed is caused by the errors in the PER of the RNs around the SN. Since the PER is calculated from the BER of a received information packet, the calculated and actual PERs for the RNs may differ somewhat due to the movement of the SN. In the proposed method, since RNs are selected in descending order of their PER, the PERs for some RNs are improved when the SN actually sends packets. Conversely, the PERs for some RNs may deteriorate. Consequently, more RNs are able to successfully receive a packet than expected. Therefore, the number of packets sent by the SN decreases, and the network load is increased due to the increase of RNs receiving a packet that the SN sends.

### 4.4. Communication Performance for Movement of RNs

**Figure 21:**
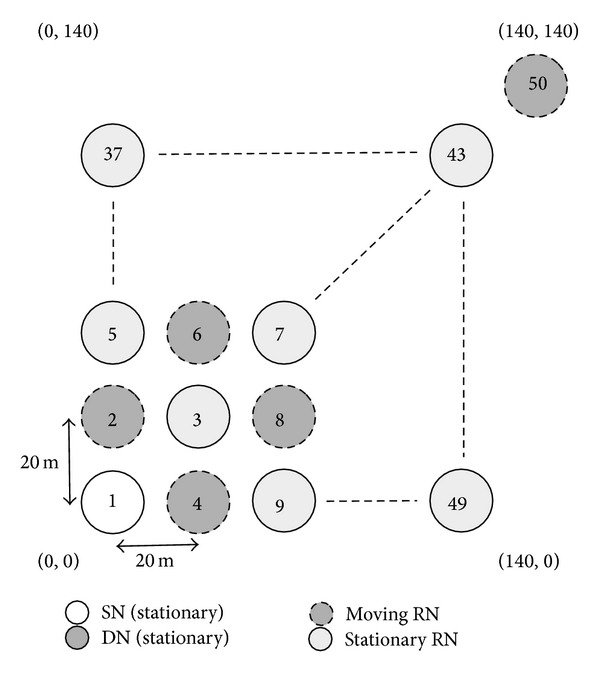
Simulation model 3 (movement of 24 RNs).

**Figure 22:**
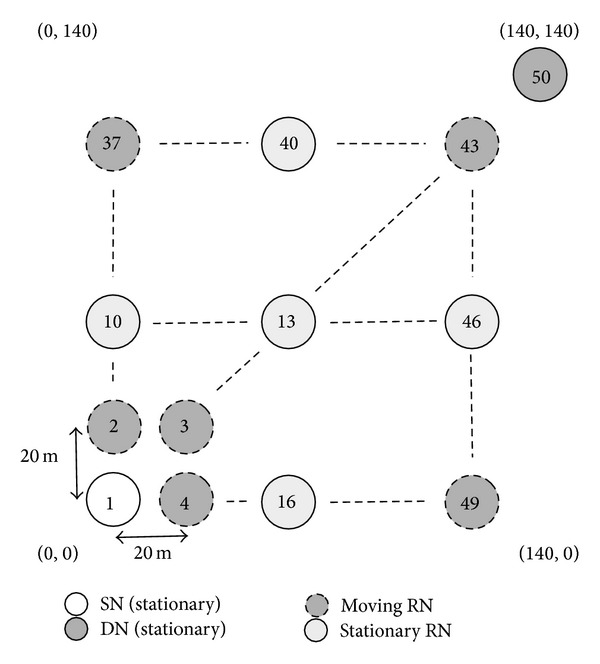
Simulation model 4 (movement of 43 RNs).

This section evaluates the impact of movement and the number of RNs on communication performance. We here employ two simulation models to describe how the movement and the number of RNs impact the communication performance of the proposed method. In the first simulation, illustrated in Figure 21, odd-numbered RNs are all stationary, whereas the remaining even-numbered RNs move randomly within the simulation area.  Figure 22 illustrates the second simulation model, where we employ more moving RNs than in the first simulation. However, when all RNs move randomly, the communication could possibly fail due to there being no RNs in the communicable range. To avoid this problem, we then employ the five situated RNs (whose node IDs are 10, 13, 16, 40, and 46). Therefore, the first simulation has 24 moving nodes, while the second simulation has 43. In both simulation models, the SN and the DN are situated at (0, 0) and (140, 140), respectively, and we vary the speed of the moving RNs from 1 m/s to 17 m/s.

**Figure 23:**
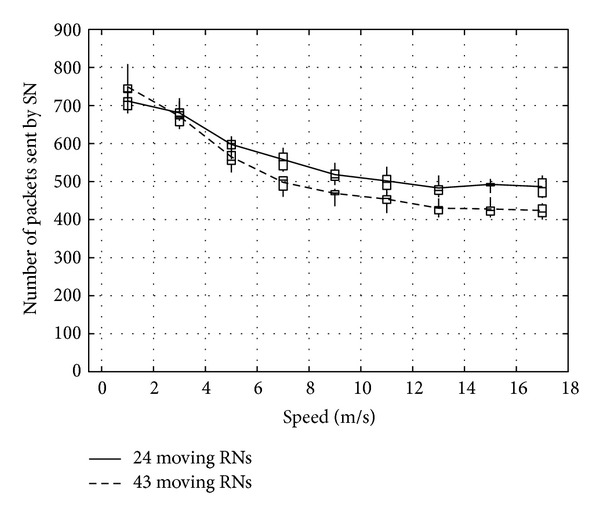
Number of packets sent by the SN.

**Figure 24:**
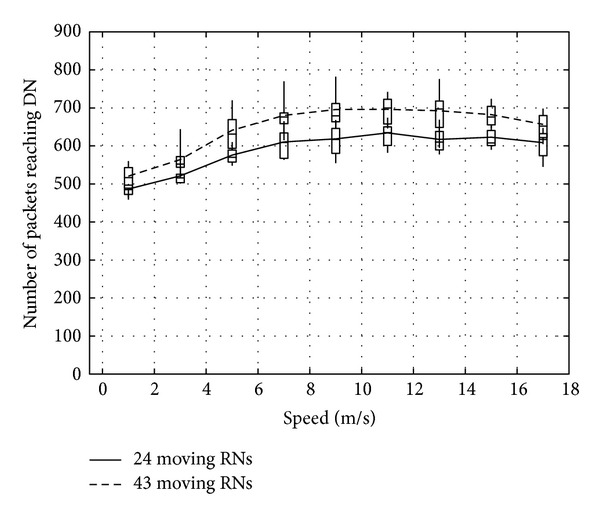
Number of packets reaching the DN.

**Figure 25:**
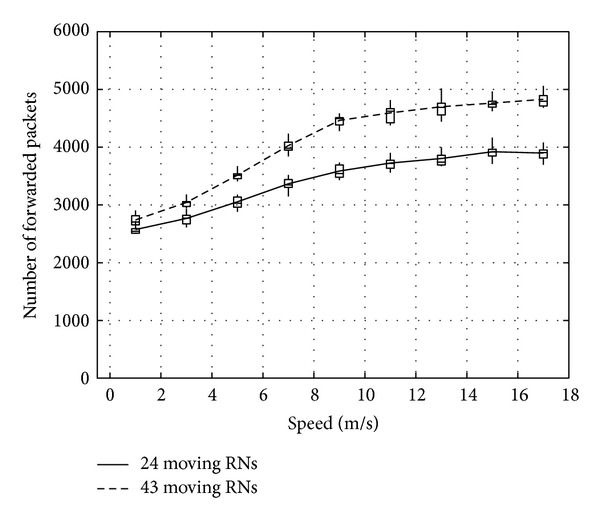
Number of packets forwarded over the network.

Figures 23, 24, and 25 show the number of packets sent by the SN, the number of packets that reached the DN, and the number of packets forwarded over the entire network, respectively. The number of packets sent by the SN decreases with the increasing movement speed of the RNs, whereas the network load increases. On the other hand, with an increase of the forwarded packets, the number of packets that reached the DN also increases. This is because, since the communication quality of some RNs improves due to movement of RNs when a packet is actually sent, more RNs successfully receive coded packets. Moreover, since higher movement speed makes it difficult to obtain appropriate location information, the number of forwarded packets over the entire network increases when the number of moving nodes is larger and their movement speed is higher.

### 4.5. Communication Performance for Movement of SN, RNs, and DN

**Figure 26:**
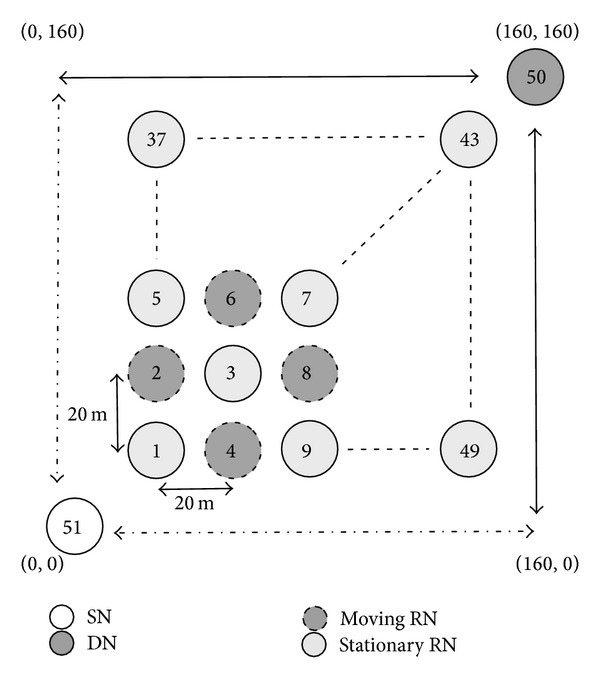
Simulation model 5 (movement of SN, DN, and RNs).

This section investigates how the movement of the SN, the DN, and the RNs impacts communication performance. As illustrated in Figure 26, the simulation model employs 51 nodes. The SN of node 51 communicates with the DN of node 50, and they continue to move at 1 m/s according to the following directions (the SN: (0, 0) → (160, 0) → (0, 0) → (0, 160) → (0, 0); the DN: (160, 160) → (0, 160) → (160, 160) → (160, 0) → (160, 160)). Odd-numbered RNs are all stationary, whereas even-numbered RNs move randomly within an area of 160 m by 160 m. We then vary the speed of the moving RNs from 1 m/s to 17 m/s.

**Figure 27:**
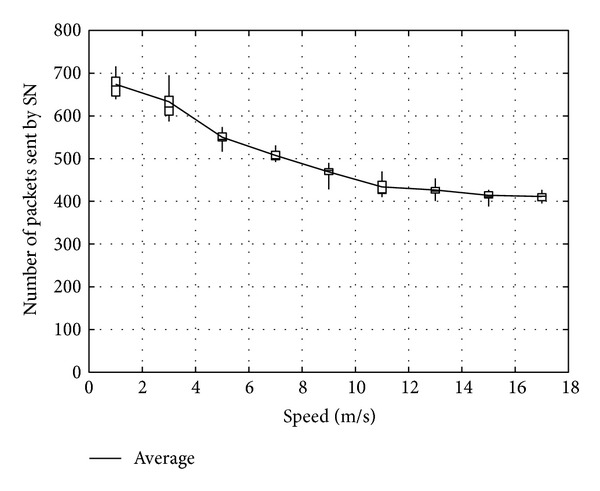
Number of packets sent by the SN.

**Figure 28:**
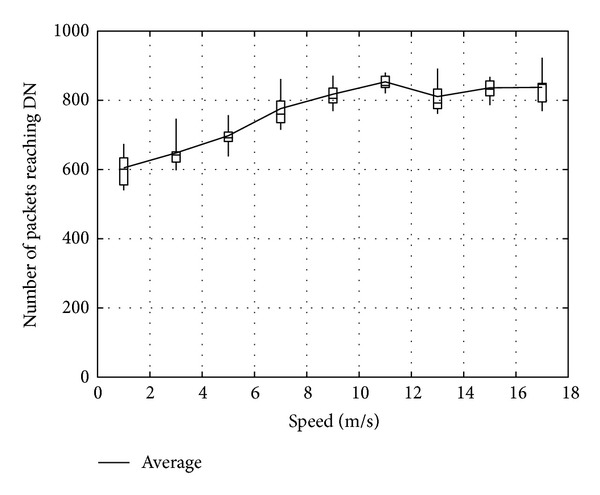
Number of packets reaching the DN.

**Figure 29:**
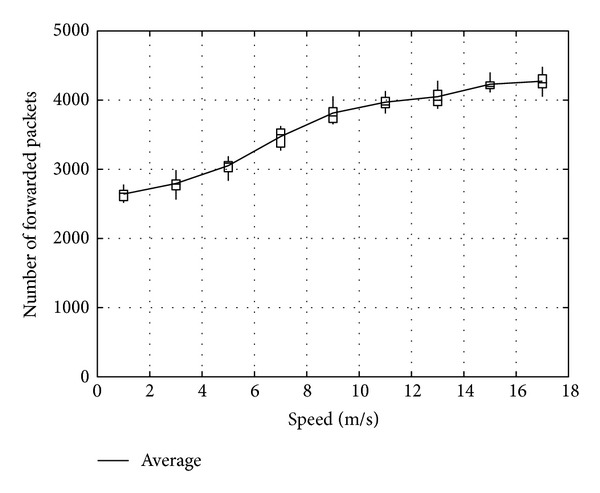
Number of packets forwarded over the network.

Figures 27, 28, and 29 show the number of packets sent by the SN, the number of packets that reached the DN, and the number of packets forwarded over the entire network, respectively. From the results, we can see that there results have almost the same features as the foregoing results.

## 5. Conclusion

In the present paper, we proposed a data delivery method based on the NNs' information in a MANET. To enhance reliable communication, the proposed method first employs opportunistic routing and network coding, in the same manner that MORE does. In addition, the proposed method uses the location information and the transmission probability of its own NNs to efficiently deliver packets to the DN. That is, in the proposed method, all of the nodes can hold the latest NNs' information within two hops by updating periodically, and a packet can then be forwarded by RNs that are appropriately selected based on the NNs' information hop-by-hop. Moreover, even if an SN sends a coded packet including erroneous location information about the DN, the packet has a fair possibility of reaching the DN because RNs within two hops from the DN can update the location information of the DN in the coded packet. Thus, the proposed method can deliver data without having to share the communication quality of each node over the entire network. We further demonstrated the communication performance of the proposed method through simulation experiments. The results of these experiments revealed that the proposed method provides better performance than MORE and the proposed method without DN's location update by RNs in a MANET environment. Notably, the proposed method can achieve efficient delivery with low network load when the movement speed is relatively slow.

We demonstrated the basic communication performance of the proposed method in grid simulation models. However, to mimic a more realistic environment, additional evaluations with changing scenarios will be required in the future. We also need to study the case in which different multiple flows exist, that is, the combination of an intraflow network coding and an interflow network coding. Although in this study we did not consider how to discover the DN to start communication in a MANET, this will be investigated in a future study.

## References

[1] Chachulski S, Jennings M, Katti S, Katabi D Trading structure for randomness in wireless opportunistic routing.

[2] Biswas S, Morris R (2005). ExOR: opportunistic multi-hop routing for wireless networks. *SIGCOMM Computer Communication Review*.

[3] Ahlswede R, Cai N, Li S-YR, Yeung RW (2000). Network information flow. *IEEE Transactions on Information Theory*.

[4] Perkins CE, Belding-Royer EM, Das SR Ad hoc On-Demand Distance Vector (AODV) Routing.

[5] Johnson DB, Maltz DA, Hu YC The Dynamic Source Routing Protocol (DSR) for Mobile Ad Hoc Networks for IPv4.

[6] Clausen TH, Jacquet P Optimized Link State Routing Protocol (OLSR).

[7] Boukerche A, Turgut B, Aydin N, Ahmad MZ, Bölöni L, Turgut D (2011). Routing protocols in ad hoc networks: a survey. *Computer Networks*.

[8] Yuan Y, Yang H, Wong SHY, Lu S, Arbaugh W ROMER: resilient opportunistic mesh routing for wireless mesh networks.

[9] Rozner E, Seshadri J, Mehta Y, Qiu L (2009). SOAR: simple opportunistic adaptive routing protocol for wireless mesh networks. *IEEE Transactions on Mobile Computing*.

[10] Katti S, Rahul H, Hu W, Katabi D, Medard M, Crowcroft J (2008). XORs in the air: practical wireless network coding. *IEEE/ACM Transactions on Networking*.

[11] Park J-S, Gerla M, Lun DS, Yi Y, Médard M (2006). CodeCast: a network-coding-based ad hoc multicast protocol. *IEEE Wireless Communications*.

[12] Mauve M, Widmer J, Hartenstein H (2001). A survey on position-based routing in mobile ad hoc networks. *IEEE Network*.

[13] Zhi N, Jing L, Botong L, Hanchun L, Youyun X A relay node selection technique for opportunistic routing in mobile ad hoc networks.

[14] Yang S, Zhong F, Yeo CK, Lee BS, Boleng J Position based opportunistic routing for robust data delivery in MANETs.

[15] De Couto DSJ, Aguayo D, Bicket J, Morris R (2005). A high-throughput path metric for multi-hop wireless routing. *Wireless Networks*.

[16] http://www.scalable-networks.com/products/qualnet/.

